# Longitudinal clinical and biomarker characteristics of non-manifesting *LRRK2* G2019S carriers in the PPMI cohort

**DOI:** 10.1038/s41531-022-00404-w

**Published:** 2022-10-22

**Authors:** Tanya Simuni, Kalpana Merchant, Michael C. Brumm, Hyunkeun Cho, Chelsea Caspell-Garcia, Christopher S. Coffey, Lana M. Chahine, Roy N. Alcalay, Kelly Nudelman, Tatiana Foroud, Brit Mollenhauer, Andrew Siderowf, Caroline Tanner, Hirotaka Iwaki, Todd Sherer, Kenneth Marek, Parkinson’s Progression Marker Initiative Authors, Andrew Siderowf, John Seibyl, Christopher Coffey, Duygu Tosun-Turgut, Leslie M. Shaw, John Q. Trojanowski, Andrew Singleton, Karl Kieburtz, Arthur Toga, Brit Mollenhauer, Douglas Galasko, Werner Poewe, Tatiana Foroud, Kathleen Poston, Susan Bressman, Alyssa Reimer, Vanessa Arnedo, Adrienne Clark, Mark Frasier, Catherine Kopil, Sohini Chowdhury, Cynthia Casaceli, Ray Dorsey, Renee Wilson, Sugi Mahes, John Seibyl, Christina Salerno, Monica Ahrens, Michael Brumm, Hyunkeun Ryan Cho, Janel Fedler, David-Erick LaFontant, Ryan Kurth, Karen Crawford, Paola Casalin, Giulia Malferrari, Mali Gani Weisz, Avi Orr-Urtreger, John Trojanowski, Leslie Shaw, Thomas Montine, Chris Baglieri, Amanda Christini, David Russell, Nabila Dahodwala, Nir Giladi, Stewart Factor, Penelope Hogarth, David Standaert, Robert Hauser, Joseph Jankovic, Marie Saint-Hilaire, Irene Richard, David Shprecher, Hubert Fernandez, Katrina Brockmann, Liana Rosenthal, Paolo Barone, Alberto Espayc, Dominic Rowe, Karen Marder, Anthony Santiago, Susan Bressman, Shu-Ching Hu, Stuart Isaacson, Jean-Christophe Corvol, Javiar Ruiz Martinez, Eduardo Tolosa, Yen Tai, Marios Politis, Debra Smejdir, Linda Rees, Karen Williams, Farah Kausar, Karen Williams, Whitney Richardson, Diana Willeke, Shawnees Peacock, Barbara Sommerfeld, Alison Freed, Katrina Wakeman, Courtney Blair, Stephanie Guthrie, Leigh Harrell, Christine Hunter, Cathi-Ann Thomas, Raymond James, Grace Zimmerman, Victoria Brown, Jennifer Mule, Ella Hilt, Kori Ribb, Susan Ainscough, Misty Wethington, Madelaine Ranola, Helen Mejia Santana, Juliana Moreno, Deborah Raymond, Krista Speketer, Lisbeth Carvajal, Stephanie Carvalo, Ioana Croitoru, Alicia Garrido, Laura Marie Payne, Veena Viswanth, Lawrence Severt, Maurizio Facheris, Holly Soares, Mark A. Mintun, Jesse Cedarbaum, Peggy Taylor, Kevin Biglan, Emily Vandenbroucke, Zulfiqar Haider Sheikh, Baris Bingol, Tanya Fischer, Pablo Sardi, Remi Forrat, Alastair Reith, Jan Egebjerg, Gabrielle Ahlberg Hillert, Barbara Saba, Chris Min, Robert Umek, Joe Mather, Susan De Santi, Anke Post, Frank Boess, Kirsten Taylor, Igor Grachev, Andreja Avbersek, Pierandrea Muglia, Kaplana Merchant, Johannes Tauscher

**Affiliations:** 1 Northwestern University Feinberg School of Medicine, Chicago, USA; 2 The University of Iowa, Iowa, USA; 3 University of Pittsburgh, Pittsburgh, USA; 4 Neurological Institute, Tel Aviv Sourasky Medical Center, Tel Aviv, Israel; 5 Department of Neurology, Columbia University, New York, NY USA; 6 Indiana University School of Medicine, Indianapolis, USA; 7 University Medical Center Goettingen Göttingen, Göttingen, Germany; 8 The University of Pennsylvania, Pennsylvania, USA; 9 University of California San Francisco, San Francisco, USA; 10 National Institute on Aging, Bethesda, USA; 11 The Michael J. Fox Foundation for Parkinson’s Research, New York, USA; 12 Institute for Neurodegenerative Disorders New York, New York, USA; 13 University of Pennsylvania, Philadelphia, PA USA; 14 Laboratory of Neuroimaging (LONI), University of Southern California, Los Angeles, CA USA; 15 Indiana University, Indianapolis, IN USA; 16 Paracelsus-Elena Klinik, Kassel, Germany; 17 University of California, San Diego, CA USA; 18 Innsbruck Medical University, Innsbruck, Austria; 19 University of Rochester, Rochester, NY USA; 20 Stanford University, Stanford, CA USA; 21 Mount Sinai, New York, NY USA; 22 Clinical Trials Coordination Center, University of Rochester, Rochester, NY USA; 23 BioRep, Milan, Italy; 24 Tel Aviv Medical Center, Tel Aviv, Israel; 25 Blackfynn, Philadelphia, PA USA; 26 Emory University of Medicine, Atlanta, GA USA; 27 Oregon Health and Science University, Portland, OR USA; 28 University of Alabama at Birmingham, Birmingham, AL USA; 29 University of South Florida, Tampa, FL USA; 30 Baylor College of Medicine, Houston, TX USA; 31 Boston University, Boston, MA USA; 32 Banner Research Institute, Sun City, AZ USA; 33 Cleveland Clinic, Cleveland, OH USA; 34 University of Tuebingen, Tuebingen, Germany; 35 Johns Hopkins University, Baltimore, MD USA; 36 University of Salerno, Salerno, Italy; 37 University of Cincinnati, Cincinnati, OH USA; 38 Macquarie University, Sydney, Australia; 39 Columbia University, New York, NY USA; 40 The Parkinson’s Institute, Sunnyvale, CA USA; 41 Beth Israel Medical Center, New York, NY USA; 42 University of Washington, Seattle, WA USA; 43 Parkinson’s Disease and Movement Disorders Center of Boca Raton, Boca Raton, FL USA; 44 Hospital Pitie-Salpetriere, Paris, France; 45 Hospital Donostia, San Sebastian, Spain; 46 Hospital Clinic de Barcelona, Barcelona, Spain; 47 Imperial College London, London, UK; 48 King’s College London, London, UK; 49 Allergan, Dublin, Ireland; 50 Abbvie, North Chicago, IL USA; 51 Avid Radiopharmaceuticals, Inc, Philadelphia, PA USA; 52 Biogen Idec, Cambridge, MA USA; 53 BioLegend, Dedham, MA USA; 54 Eli Lilly and Company, Indianapolis, IN USA; 55 GE Healthcare, Princeton, NJ USA; 56 Genentech, San Francisco, CA USA; 57 Genzyme Sanofi, Cambridge, MA USA; 58 GlaxoSmithKline, Brentford, UK; 59 H. Lundbeck A/S, Copenhagen, Denmark; 60 Institut de Recherches Internationales Servier, Neuilly-sur-Seine, France; 61 Merck and Co, Kenilworth, NJ USA; 62 Meso Scale Diagnostics, Rockville, MD USA; 63 Pfizer Inc, Cambridge, MA USA; 64 Piramal Group, Mumbai, India; 65 F. Hoffmann-La Roche Limited, Basel, Switzerland; 66 Teva Pharmaceutical Industries, Petah Tikva, Israel; 67 UCB Pharma, Brussel Belgium, Belgium; 68 TransThera Consulting, Portland, OR USA; 69 Takeda, Osaka, Japan

**Keywords:** Parkinson's disease, Movement disorders, Neurological disorders, Biomarkers

## Abstract

We examined 2-year longitudinal change in clinical features and biomarkers in *LRRK2* non-manifesting carriers (NMCs) versus healthy controls (HCs) enrolled in the Parkinson’s Progression Markers Initiative (PPMI). We analyzed 2-year longitudinal data from 176 *LRRK2* G2019S NMCs and 185 HCs. All participants were assessed annually with comprehensive motor and non-motor scales, dopamine transporter (DAT) imaging, and biofluid biomarkers. The latter included cerebrospinal fluid (CSF) Abeta, total tau and phospho-tau; serum urate and neurofilament light chain (NfL); and urine bis(monoacylglycerol) phosphate (BMP). At baseline, *LRRK2* G2019S NMCs had a mean (SD) age of 62 (7.7) years and were 56% female. 13% had DAT deficit (defined as <65% of age/sex-expected lowest putamen SBR) and 11% had hyposmia (defined as ≤15th percentile for age and sex). Only 5 of 176 *LRRK2* NMCs developed PD during follow-up. Although NMCs scored significantly worse on numerous clinical scales at baseline than HCs, there was no longitudinal change in any clinical measures over 2 years or in DAT binding. There were no longitudinal differences in CSF and serum biomarkers between NMCs and HCs. Urinary BMP was significantly elevated in NMCs at all time points but did not change longitudinally. Neither baseline biofluid biomarkers nor the presence of DAT deficit correlated with 2-year change in clinical outcomes. We observed no significant 2-year longitudinal change in clinical or biomarker measures in *LRRK2* G2019S NMCs in this large, well-characterized cohort even in the participants with baseline DAT deficit. These findings highlight the essential need for further enrichment biomarker discovery in addition to DAT deficit and longer follow-up to enable the selection of NMCs at the highest risk for conversion to enable future prevention clinical trials.

## Introduction

Slowing or preventing the progression of Parkinson’s disease (PD) remains a major unmet goal. To this end, therapeutic interventions that target pathogenic mechanisms in genetically-defined subgroups offer an attractive strategy. Along with *GBA*, pathogenic variants in the gene encoding leucine-rich repeat kinase 2 (*LRRK2*) represent the most common genetic cause of PD, with G2019S being the most prevalent pathogenic variant. Not surprisingly, a pipeline of *LRRK2*-targeted therapies is in clinical development^1^. Although current studies target populations with manifest PD, intervention in the pre-manifest phase of the disease offers an opportunity for disease prevention. Thus, non-manifesting carriers (NMCs) of *LRRK2* pathogenic variants are a unique prodromal PD population for disease prevention studies. However, to enable such trials two major knowledge gaps must be addressed. First, establish the trajectory of clinical and biomarker changes during the prodromal stage in *LRRK2* NMCs and second, elucidate clinical outcomes and/or biomarkers that can identify the *LRRK2* NMC subgroup at the highest risk of developing PD. The latter is especially critical because *LRRK2* pathogenic variants have a low, variable (25–40%) and age-modified lifelong penetrance^2^. Although there is a growing body of literature examining motor, non-motor and biomarker characteristics of *LRRK2* NMCs^3–5^, there are limited longitudinal data from large prospective studies. We have previously reported the presence of subtle motor and non-motor signs of PD in *LRRK2* NMCs compared to healthy controls (HCs) enrolled in the Parkinson’s Progression Markers Initiative (PPMI) study in cross-sectional analysis but indicated that longitudinal data will be essential to confirm the findings and establish trajectory and baseline predictors of progression in that cohort^6^.

The objectives of the present analysis were to: (1) systematically evaluate 2-year longitudinal change in clinical and dopamine transporter (123-I Ioflupane (DatScan®)) imaging (DATscan) characteristics of *LRRK2* NMCs compared with HCs enrolled in the PPMI study; (2) report for the first time baseline and longitudinal data on an array of biofluid biomarkers; and (3) assess baseline predictors of progression.

## Results

### Baseline demographic and DAT imaging characteristics

176 *LRRK2* G2019S NMCs and 185 HCs were included in the analysis. Five NMCs and no HCs developed PD in the course of the study (Table 1). Baseline demographics, PD family history, presence of hyposmia, presence of a DAT deficit, and percent of participants meeting MDS prodromal research criteria above 80% are presented in Table 1. The demographic characteristics and comparison to HCs are consistent with our previous report^6^. Considering that hyposmia is a significant prodromal risk for PD in general, University of Pennsylvania Smell Identification test (UPSIT) scores are presented as raw values and by different percentile cut offs. There was no difference in severe hyposmia (≤15^th^ percentile for age and sex) between NMCs and HCs but a higher percent of NMCs (41% vs 27%) had moderately reduced sense of smell. A minority (13%) of NMCs had a DAT deficit, which was not significantly different (*p* = 0.0546) compared to HCs. Only 7% of NMCs met MDS prodromal criteria.

**Table 1:** Demographics and baseline characteristics.

	Group		*p* values	*LRRK2* G2019S converters
	Healthy controls (*N* = 185)	*LRRK2* G2019S carriers (*N* = 176)	*LRRK2* G2019S *vs* healthy controls	Consensus Committee (*N* = 5)
Sex (female)	66 (36%)	99 (56%)	<0.0001	5 (100%)
Age, mean (SD; range)	61.0 (11.1; 30–83)	62.0 (7.7; 45–81)	0.3225	70.0 (12.5; 53–81)
Education (≤12 years)	27 (15%)	26 (15%)	0.9619	1 (20%)
Ethnicity (Hispanic/Latino)	2 (1%)	15 (9%)	0.0008	0 (0%)
Race (White)	172 (93%)	171 (98%)	0.0340	5 (100%)
Family history of PD (first-degree)	0 (0%)	152 (86%)	<0.0001	5 (100%)
UPSIT raw score, mean (SD; range)	34.2 (4.4; 16–40)	33.1 (4.3; 14–40)	0.0261	30.6 (7.0; 19–36)
UPSIT percentile			0.0055	
≤15th	17 (9%)	20 (11%)		2 (40%)
16th-50th	50 (27%)	73 (41%)		0 (0%)
>50th	118 (64%)	83 (47%)		3 (60%)
DAT deficit	12 (7%)	21 (13%)	0.0546	4 (100%)
Missing DAT results	1	8		1
Caudate SBR	2.99 (0.61)	2.97 (0.57)	0.5857	2.24 (0.37)
Putamen SBR	2.15 (0.54)	2.09 (0.51)	0.2960	1.09 (0.30)
Striatum SBR	2.57 (0.55)	2.53 (0.52)	0.4178	1.66 (0.31)
MDS prodromal criteria (>80%)	1 (1%)	12 (7%)	0.0014	3 (60%)

Data are mean (SD) or n (%) unless otherwise stated. DAT deficit defined as <65% age/sex-expected lowest putamen SBR. Ethnicity and race were missing for one *LRRK2* G2019S subject. p values for SBR measures were adjusted for age and sex using inverse probability weighting; all other p values were found using chi-square and t-tests. *LRRK2* G2019S converters reflect a subset of the overall *LRRK2* G2019S group. *UPSIT* University of Pennsylvania Smell Identification Test, *DAT* dopamine transporter, *SBR* specific binding ratio, *MDS* International Parkinson and Movement Disorder Society.

### Clinical and biological characteristics of the participants who developed PD (“phenoconverters”)

5 NMCs developed clinically defined PD at the time of data analysis. Baseline demographic characteristics of these participants are presented in Table 1 and longitudinal clinical characteristics compared to the rest of the cohort are presented in Suppl. Table 4. Considering the small sample size, no statistical comparisons were performed. These participants were older and all females (Table 1). As expected, they had higher baseline and longitudinal Movement Disorders Society Unified Parkinson’s Disease Rating Scale (MDS-UPDRS) total and subscores. 4 of 5 had baseline DAT data available and all showed DAT deficit (Table 1). Only 2 of 5 had hyposmia and 3 of 5 met MDS prodromal criteria at baseline. The diagnosis of PD was made within 12 months of baseline in 4 participants and at 48 months in 1. None of the participants developed other neurodegenerative conditions.

## Longitudinal data

### Clinical characteristics

Longitudinal change in a wide range of clinical characteristics in NMCs at the group level were compared to HCs (Table 2). Consistent with our previous report^6^, at baseline, NMCs scored significantly worse than HCs on numerous clinical scales. However, there was no significant longitudinal change in any of these measures over 2 years. Since DAT deficit signifies the presence of presynaptic striatal dopaminergic deficiency, analyses of the longitudinal change in clinical characteristics in NMCs subdivided by DAT deficit (<65% versus above) (Table 3) was performed. There was no significant group by time effect, indicating that there was no difference between groups in the longitudinal change from baseline to follow-up; though it is noteworthy that few participants had a baseline DAT deficit (13%). We repeated the same analysis dichotomizing the carriers on the baseline degree of hyposmia (≤15th percentile or above) and similarly did not observe a significant group by time effect (Suppl. Table 1).

**Table 2:** Change in clinical characteristics among *LRRK2* G2019S carriers *vs* healthy controls.

	*LRRK2* G2019S carriers			Healthy controls			*p* value
	Baseline (*N* = 176)	Year 1 (*N* = 172)	Year 2 (*N* = 140)	Baseline (*N* = 185)	Year 1 (*N* = 182)	Year 2 (*N* = 172)	Group × time effect
MDS-UPDRS total score	8.9 (7.4)	9.6 (8.4)	9.4 (9.2)	4.6 (4.4)	5.3 (5.2)	5.3 (4.9)	0.6519
MDS-UPDRS Part I	4.8 (3.8)	5.0 (4.1)	4.7 (4.0)	3.0 (2.9)	3.2 (3.2)	3.2 (3.0)	0.1848
MDS-UPDRS Part II	1.1 (2.2)	1.4 (2.7)	1.4 (2.6)	0.4 (1.0)	0.4 (1.1)	0.6 (1.3)	0.0194
MDS-UPDRS Part III	3.1 (4.1)	3.2 (4.1)	3.2 (5.0)	1.2 (2.2)	1.6 (2.8)	1.5 (2.8)	0.5018
MOCA total score, mean (SD; range)	26.8 (2.4; 18–30)	27.0 (2.5; 16–30)	26.9 (2.4; 20–30)	28.2 (1.1; 26–30)*	27.2 (2.2; 20–30)	27.2 (2.4; 21–30)	0.0044
GDS-15 total score	1.7 (2.4)	1.5 (2.1)	1.7 (2.3)	1.3 (2.1)	1.4 (2.4)	1.2 (1.9)	0.4104
SCOPA-AUT total score	8.4 (6.1)	8.8 (6.6)	8.6 (6.2)	5.8 (3.8)	5.9 (4.5)	6.0 (4.2)	0.0458
Orthostatic systolic blood pressure drop	0.1 (11.0)	−0.9 (11.2)	−0.5 (10.7)	2.1 (12.3)	1.7 (10.5)	1.0 (11.3)	0.0702
Orthostatic diastolic blood pressure drop	−3.7 (7.6)	−4.6 (7.6)	−3.2 (7.6)	−3.5 (8.4)	−2.5 (7.4)	−3.5 (8.0)	0.1580
State trait anxiety score	61.3 (16.7)	60.2 (16.6)	60.4 (16.8)	57.0 (14.2)	56.3 (16.8)	55.9 (14.5)	0.2646
QUIP (≥1 disorder)	46 (27%)	45 (27%)	44 (32%)	36 (19%)	37 (20%)	29 (17%)	0.0713
Epworth sleepiness scale (≥ 10)	18 (10%)	17 (10%)	14 (10%)	20 (11%)	19 (10%)	23 (13%)	0.5677
RBDSQ (≥5)	38 (22%)	35 (21%)	27 (20%)	36 (19%)	35 (19%)	29 (17%)	0.6721

Data are mean (SD) or *n* (%) unless otherwise stated. Five or fewer participants per group missed any one assessment except for *LRRK2* G2019S carriers at year 1 (six missed the MDS-UPDRS total score and RBDSQ) and year 2 (10 missed the SCOPA-AUT; six missed the STAI). *p* values were found using generalized estimating equations (with inverse probability weighting to adjust for age and sex) modeling the change from baseline at follow-up (continuous outcomes) or the binary response at follow-up (categorical outcomes) while adjusting for the baseline value. Significance level for comparisons is *p* < 0.0038 (after Bonferroni correction).

*Exclusion criteria for healthy controls included a baseline MOCA score < 27, but a waiver was granted to one individual with a baseline score of 26.

*MDS-UPDRS* Movement Disorder Society Unified Parkinson’s Disease Rating Scale. *MOCA* Montreal Cognitive Assessment. *GDS-15* Geriatric Depression Scale (15-item). *SCOPA-AUT* Scales for Outcomes in Parkinson’s Disease-Autonomic. *QUIP* Questionnaire for Impulsive-Compulsive Disorders in Parkinson’s Disease. *RBDSQ* REM Sleep Behavior Disorder Screening Questionnaire.

**Table 3:** Change in clinical characteristics among *LRRK2* G2019S carriers with *vs* without baseline DAT deficit.

	*LRRK2* G2019S carriers: baseline DAT deficit			*LRRK2* G2019S carriers: no baseline DAT deficit			*p* value
	Baseline (*N* = 21)	Year 1 (*N* = 21)	Year 2 (*N* = 15)	Baseline (*N* = 147)	Year 1 (*N* = 144)	Year 2 (*N* = 118)	Group × time effect
MDS-UPDRS total score	10.8 (10.1)	12.5 (10.8)	15.1 (14.1)	8.6 (6.9)	9.1 (7.8)	8.8 (8.1)	0.1039
MDS-UPDRS Part I	5.1 (4.2)	5.6 (4.3)	5.2 (3.2)	4.7 (3.7)	5.0 (4.0)	4.8 (4.2)	0.6761
MDS-UPDRS Part II	2.3 (3.5)	3.1 (4.4)	2.6 (2.8)	0.9 (1.9)	1.2 (2.3)	1.2 (2.5)	0.1199
MDS-UPDRS Part III	4.3 (5.3)	5.4 (6.3)	7.4 (10.4)	2.9 (3.8)	2.9 (3.6)	2.8 (3.7)	0.0578
MOCA total score, mean (SD; range)	26.6 (2.2; 22–30)	26.8 (2.4; 23–30)	26.4 (3.1; 20–30)	26.9 (2.5; 18–30)	27.1 (2.5; 16–30)	26.9 (2.3; 21–30)	0.7475
GDS-15 total score	2.0 (3.0)	1.7 (2.0)	1.9 (2.6)	1.6 (2.2)	1.4 (1.9)	1.6 (2.0)	0.9898
SCOPA-AUT total score	9.7 (7.1)	10.2 (7.8)	11.2 (5.8)	8.1 (5.8)	8.5 (6.5)	8.2 (6.2)	0.1808
Orthostatic systolic blood pressure drop	−4.5 (9.9)	−3.3 (9.6)	−4.3 (13.0)	0.7 (10.8)	−0.6 (11.4)	0.2 (10.5)	0.1495
Orthostatic diastolic blood pressure drop	−6.3 (5.9)	−6.5 (7.0)	−6.9 (6.2)	−3.7 (7.7)	−4.3 (7.8)	−3.0 (7.4)	0.0746
State trait anxiety score	64.0 (17.9)	58.2 (14.1)	58.4 (16.4)	60.9 (16.4)	60.2 (16.4)	60.3 (15.8)	0.1896
QUIP ( ≥ 1 disorder)	4 (20%)	5 (26%)	3 (21%)	39 (27%)	39 (28%)	38 (33%)	0.6539
Epworth sleepiness scale (≥10)	2 (10%)	2 (10%)	1 (7%)	16 (11%)	14 (10%)	12 (10%)	0.8113
RBDSQ ( ≥ 5)	3 (15%)	5 (25%)	2 (14%)	35 (24%)	29 (21%)	25 (22%)	0.4639

Data are mean (SD) or *n* (%) unless otherwise stated. DAT deficit defined as <65% age/sex-expected lowest putamen specific binding ratio (eight *LRRK2* G2019S carriers are excluded due to missing baseline DAT data). Among subgroup with baseline DAT deficit, two or fewer participants missed any one assessment; among subgroup without baseline DAT deficit, five or fewer participants missed any one assessment except for the SCOPA-AUT at year 2 (seven missing values). *p* values were found using generalized estimating equations modeling the change from baseline at follow-up (continuous outcomes) or the binary response at follow-up (categorical outcomes) while adjusting for the baseline value. Significance level for comparisons is *p* < 0.0038 (after Bonferroni correction).

*DAT* dopamine transporter, *MDS-UPDRS* Movement Disorder Society Unified Parkinson’s Disease Rating Scale, *MOCA* Montreal Cognitive Assessment, *GDS-15* Geriatric Depression Scale (15-item), *SCOPA-AUT* Scales for Outcomes in Parkinson’s Disease-Autonomic, *QUIP* Questionnaire for Impulsive-Compulsive Disorders in Parkinson’s Disease, *RBDSQ* REM Sleep Behavior Disorder Screening Questionnaire.

### DAT imaging

There also was no significant longitudinal decline in caudate (*p* = 0.1929), putamen (*p* = 0.0865) or striatum (*p* = 0.0910) specific binding ratios (SBRs) in NMCs. There was a trend but no significant difference in SBR values at 2-year follow-up in NMCs compared to HCs at baseline after adjusting for multiple comparisons (Suppl. Table 2).

### Biofluid biomarker characteristics

We analysed biofluid biomarkers in cerebrospinal fluid (CSF) Abeta, total tau and phospho-tau; and serum urate and neurofilament light chain (NfL) (Table 4). There were no differences in any of these biomarkers between NMCs and HC at baseline and no difference in longitudinal changes between groups. Of note, CSF AD biomarkers are presented as median (range) values as well as above or below cut-offs established in AD literature for the sake of comparison and cross referencing^7,8^. We also ran analyses of urine bis(monoacylglycerol) phosphate (BMP) as a biomarker of lysosomal dysfunction previously shown to be elevated in *LRRK2* cohorts^9^. Consistent with our published data, there was significant baseline elevation of all tested urinary BMP species in NMCs compared to HCs (Table 4). Relative to HCs, levels for NMCs were higher by 3.8-fold (95% CI 3.1–4.5) for total di-18:1-BMP, 6.1-fold (95% CI 5.3–7.1) for total di-18:1-BMP, and 7.4-fold (6.4–8.6) for 2,2′-di-22:6 BMP. However, there was no longitudinal change in any BMP species in NMCs (Suppl Table 3). CSF alpha-synuclein measures were not available, as they are still under analysis. The polygenic risk score did not differ between NMCs and HCs.

**Table 4:** Baseline and longitudinal biofluids among *LRRK2* G2019S carriers *vs* healthy controls.

	LRRK2 G2019S carriers			Healthy controls			*p* values	
	Baseline (*N* = 176)	Year 1 (*N* = 172)	Year 2 (*N* = 140)	Baseline (*N* = 185)	Year 1 (*N* = 182)	Year 2 (*N* = 172)	Group effect	Group × time effect
Polygenic risk score	0.11 (0.97)	—	—	−0.10 (1.02)	—	—	0.3806	—
CSF Abeta, median (min, max)	924 (219, 1475)	952 (200, 1475)	944 (200, 1475)	937 (239, 1475)	999 (312, 1475)	948 (249, 1475)		
Low (<683 pg/mL)	29 (24%)	29 (24%)	18 (21%)	44 (28%)	33 (22%)	33 (25%)	0.5290	0.4986
Missing	53	53	56	27	35	41		
CSF tTau, median (min, max)	167 (80, 506)	162 (80, 631)	169 (80, 498)	172 (80, 581)	175 (80, 600)	178 (80, 620)		
High (>266 pg/mL)	12 (10%)	12 (10%)	8 (10%)	20 (12%)	25 (17%)	23 (17%)	0.1736	0.1030
CSF pTau, median (min, max)	14.2 (8.0, 46.1)	14.1 (8.0, 57.0)	14.0 (8.0, 44.3)	14.9 (8.0, 73.6)	15.2 (8.0, 80.1)	14.9 (8.0, 80.5)		
High (>24 pg/mL)	8 (7%)	10 (8%)	5 (6%)	21 (13%)	24 (16%)	23 (17%)	0.0304	0.0470
Missing tTau and pTau	53	53	56	24	32	39		
Serum urate	316.3 (83.0)	308.9 (80.1)	311.4 (75.0)	322.3 (78.7)	321.2 (74.9)	326.0 (79.7)	0.1336	0.0445
Missing	35	36	33	8	18	13		
Serum NfL	13.1 (6.6)	13.9 (7.0)	13.1 (5.6)	11.7 (6.6)	12.4 (7.7)	12.1 (5.7)	0.1103	0.8532
Median (min, max)	11.3 (3.5, 47.1)	12.4 (3.7, 49.9)	11.8 (3.3, 30.4)	10.5 (2.4, 51.0)	10.9 (2.3, 70.9)	10.8 (3.3, 29.8)		
Missing	58	61	63	15	28	39		
Urine total di-18:1-BMP	17.5 (13.4)	18.6 (17.0)	20.3 (17.7)	4.9 (4.0)	—	—	<0.0001*	—
Median (min, max)	14.2 (0.9, 72.0)	13.0 (2.3, 99.0)	14.8 (1.7, 76.5)	3.8 (0.4, 20.2)				
Urine total di-22:6-BMP	77.3 (46.5)	81.0 (52.8)	85.5 (55.2)	13.6 (12.4)	—	—	<0.0001*	—
Median (min, max)	63.8 (10.6, 245.6)	67.2 (7.5, 289.6)	72.6 (15.8, 320.8)	10.5 (1.3, 85.9)				
Urine 2,2′-di-22:6 BMP	61.3 (40.3)	61.9 (42.5)	65.9 (42.9)	8.9 (9.1)	—	—	<0.0001*	—
Median (min, max)	49.6 (8.1, 248.2)	47.0 (2.2, 244.2)	57.1 (12.8, 252.8)	6.5 (0.6, 66.0)				
Missing BMP	33	32	54	3				

Data are mean (SD) or *n* (%) unless otherwise stated. Fifteen *LRRK2* G2019S carriers and two healthy controls were missing polygenic risk score results. Group effect p values were found using inverse probability weighting methods comparing baseline values while adjusting for age and sex (analysis of polygenic risk score also adjusted for the first five principal components of the genetic data). Group × time effect *p* values were found using generalized estimating equations (with inverse probability weighting to adjust for age and sex) modeling the change from baseline at follow-up (continuous outcomes) or the binary response at follow-up (categorical outcomes) while adjusting for the baseline value. BMP group × time effects were not assessed because healthy controls only had baseline results.

*CSF* cerebrospinal fluid. *NfL* neurofilament light chain. *BMP* bis(monoacylglycerol)phosphate.

*Significance levels for comparisons are *p* < 0.0056 for group effects and *p* < 0.01 for group × time effects (after Bonferroni correction).

### Association of biofluid biomarkers with clinical and DAT imaging characteristics

Utilizing MDS-UPDRS Part III score as a measure of evolving motor parkinsonism, none of the fluid biomarkers included in our array correlated with baseline or longitudinal change in MDS- UPDRS Part III (data not shown). We repeated the analysis looking at correlation between baseline polygenic risk score, biofluid biomarkers, and baseline DAT (mean striatum SBR). The only significant correlations were with the total di-22:6 (Spearman’s rho = −0.28; 95% CI −0.42 to −0.13) and 2,2′-di-22:6 (−0.27; −0.41 to −0.12) BMP species (Table 5). However, there was no correlation of any baseline biofluid biomarker level, including polygenic risk score and BMP, with the 2-year change in mean striatum SBR (Table 5). There was no significant effect of baseline serum urate level on change in any of the clinical characteristics (data not shown).

**Table 5:** Correlations between baseline biomarkers *vs* baseline and longitudinal change in DAT specific binding ratios among *LRRK2* G2019S carriers.

	Correlation with baseline mean striatum SBR			Correlation with 2-year percent change from baseline in mean striatum SBR		
Baseline Biomarker	N Obs	Spearman’s Rho	*p* value	N Obs	Spearman’s Rho	*p* value
Polygenic risk score	155	−0.10	0.2304	115	0.03	0.7513
Serum urate	140	0.02	0.8513	101	−0.01	0.8880
CSF Abeta (quintile)	143	−0.06	0.4876	105	0.13	0.2016
CSF tau (quintile)	143	0.02	0.7903	105	0.15	0.1296
CSF p-tau (quintile)	143	0.07	0.3942	105	0.14	0.1511
Serum NfL	135	0.13	0.1342	114	−0.03	0.7724
Urine total di-18:1-BMP	164	−0.15	0.0523	119	−0.06	0.5067
Urine total di-22:6-BMP	164	−0.28	0.0003*	119	-0.03	0.7298
Urine 2,2-di-22:6-BMP	164	−0.27	0.0005*	119	−0.04	0.6932

Correlation coefficients and *p* values were computed using Spearman partial rank-order correlations controlling for age and sex (correlations with polygenic risk score also controlled for the first five principal components of the genetic data; correlations with 2-year change in DAT also controlled for mean striatum SBR at baseline).

*DAT* dopamine transporter. *SBR* specific binding ratio. *CSF* cerebrospinal fluid. *NfL* neurofilament light chain. *BMP* bis(monoacylglycerol)phosphate. *Significance level for comparisons is *p* < 0.0056 (after Bonferroni correction).

## Discussion

We present the longitudinal clinical, DAT imaging, and biofluid biomarker data for a large *LRRK2* G2019S NMC cohort compared to HCs. To our knowledge, this is the largest comprehensive longitudinal dataset reported to date. These data prompt several novel observations that are highly relevant to future PD prevention trials.

Consistent with slow progression and low penetrance, only 5/176 carriers developed clinically defined PD. These observations highlight the essential importance of identifying clinical and biological markers for the enrichment of the NMC cohort to identify those with the greatest risk of developing PD for future therapeutic interventions. We have systematically explored a number of such predictors here.

We and others have previously reported higher prevalence of PD motor and non-motor features in *LRRK2* G2019S NMCs compared to HCs in cross-sectional studies^3–5,10–12^. However, our data do not show any significant longitudinal change in these clinical outcomes. The most likely explanation is the relatively slow progression of the phenotypic changes, consistent with incomplete penetrance of this variant and lack of risk-based enrichment in this cohort.

MDS prodromal research criteria were developed to identify participants with higher likelihood of prodromal PD^13,14^. MDS prodromal criteria have shown a wide range of positive predictive values (19–81%) in prodromal cohorts and perform best in REM sleep behavior disorder (RBD) cohorts, consistent with the fact that they are heavily weighted for the presence of idiopathic RBD^15–17^. Only 7% of our NMCs met MDS prodromal criteria at baseline, which may explain the lack of meaningful progression in our cohort. On the other hand, only 60% of the converters met the MDS prodromal criteria at baseline suggesting that other risk factors may underlie the progression to clinical PD in *LRRK2* G2019S carriers. MDS prodromal criteria have been applied in another *LRRK2* G2019S cohort and showed 47% positive predictive value over 5 years^12^. In aggregate, these findings argue that MDS criteria are less sensitive in *LRRK2* cohorts and additional variables will be important to identify the population at the highest risk of progression. These findings also highlight the essential need for biomarker enrichment of *LRRK2 G2019S* NMC cohorts to identify participants at risk for progression.

The presence of DAT deficit is the strongest biomarker predictor of progression to clinically defined PD in RBD and hyposmic prodromal cohorts^18,19^ and should be an important enrichment biomarker for genetic NMC cohorts as well. Reduction of SBR values in *LRRK2* G2019S NMCs compared to HCs was reported by other groups, though in smaller cohorts and cross-sectional observations^5,4,11,20,21^. Only 13% of the *LRRK2* G2019S NMCs had baseline DAT deficit, which is the likely explanation for the lack of significant progression using a range of outcome measures. However, there was no significant progression on any clinical measure even in the sub-group of participants with DAT deficit (Table 3). At least three possibilities may have contributed to this observation: the relatively small number of participants with DAT deficit at baseline, short observation period (2 years), or a slower progression rate in *LRRK2* G2019S NMCs compared to other prodromal cohorts. Further clarification of this observation is of essential importance for experimental therapeutics and will require longer follow-up in larger cohorts. Enrichment based on DAT deficit was not applied in the original PPMI NMC cohorts as it was essential to establish the prevalence of such in the overall study population. Current PPMI enrollment criteria include enrichment based on DAT deficit; however, the cut-off value (60–80%) will be established from the prospective longitudinal study. Our analyses here indicate a major challenge for DAT deficit-based enrichment of *LRRK2* NMC cohorts. Specifically, >80% of currently enrolled *LRRK2* G2019S NMCs will not qualify for the high-risk prodromal group if we apply the cut-off of <65% DAT deficit. Thus, additional enrichment strategies are required for the *LRRK2* NMC prodromal group.

Hyposmia is a common prodromal feature in PD and has been used for staged enrichment of prodromal cohorts^19^. However, it is less common in *LRRK2* PD and specifically in G2019S carriers^4^. Consistent with our and others’ previous reports, the prevalence of severe hyposmia in *LRRK2* G2019S NMCs was low and baseline hyposmia did not associate with longitudinal change in any clinical variables. Notably, though, baseline hyposmia was associated with higher odds of baseline DAT deficit (odds ratio=3.52, 95% CI 1.10 to 11.27).

The strongest and most novel attribute of our dataset is the richness of biomarker characterization. We structured analyses to assess if any of the tested biomarkers: (1) separated NMCs from HCs; (2) demonstrated longitudinal change; or (3) were associated with baseline or longitudinal changes in DAT or motor parkinsonian features. None of the CSF or serum biomarkers tested had significant findings in any of these analyses. Confirming our previously reported data, NMCs had significantly elevated BMP levels at baseline compared to HCs^9^. Baseline BMP levels correlated with baseline mean striatum SBRs but did not show longitudinal change and did not correlate with the 2-year change in DAT values. In a separate analysis (not shown) there was no significant difference in BMP levels between *LRRK2* NMCs and *LRRK2* with PD. As such BMP is an important biomarker to study *LRRK2*-driven pathogenic mechanisms or modulation of *LRRK2* activity by therapeutics (i.e., target modulation biomarker) but does not appear to be a biomarker of progression or risk to develop PD in prodromal individuals. CSF Abeta, total tau or p-tau levels did not differ between *LRRK2* G2019S NMCs versus HCs. These indices are of interest considering that some *LRRK2* carriers have tau rather than alpha-synuclein pathology^22^ but a direct test would require tau imaging studies in *LRRK2* pathogenic variant carriers. Polygenic risk score also was not associated with the change in DAT.

We recognize that our study has several limitations. First, we do not have data on CSF monomeric or aggregated alpha-synuclein levels since the biosamples have not been fully analyzed for these markers. However, based on previous reports, we do not expect to see a significant longitudinal change in monomeric or aggregated alpha-synuclein^23^. Alpha-synuclein seed amplification assays (SAA; also termed PMCA or RTQuIC), while still only qualitative, have a high potential to detect prodromal alpha-synuclein pathology^24,25^ and thereby represent a promising biomarker for the enrichment of an at-risk population. In the PPMI cohorts, alpha-synuclein SAAs on the CSF and skin are being conducted currently and data will be shared once available. These studies could help identify the stage at which alpha-synuclein pathology develops in prodromal participants. SAA data will be of paramount importance in all prodromal cohorts, and specifically in *LRRK2* carriers, to confirm the presence of alpha-synuclein driven pathology to select individuals appropriate for alpha-synuclein targeted interventions.

We and others have reported several *LRRK2*-associated biomarkers including blood metabolomics, urinary proteome, and inflammatory markers^26–29^. In addition, *LRRK2*-associated pharmacodynamic biomarkers have also been developed as reviewed recently^27,30^. Some of these distinguish *LRRK2* manifest and NMCs from sporadic PD and HC. However, none have been shown to correlate with longitudinal disease progression. Importantly, specific biomarkers/panels from these studies are being tested on PPMI biosamples to assess their utility as prognostic or progression markers. There are few pilot studies showing promising data on novel functional MRI sequence imaging biomarkers that will require further testing^31^. Digital biomarkers offer tremendous promise for baseline enrichment and longitudinal follow-up of the NMC cohorts^32^. These are being collected in PPMI and other cohorts and will be reported as the data become available. Lastly, our analysis was restricted to G2019S *LRRK2* carriers. While G2019S is the most common *LRRK2* pathogenic variant, it is not the only one. While we identified 16 R1441G NMCs, the sample size was too small to run any comparative analysis and considering data on difference in phenotypic characteristics and progression, we decided not to combine the analysis (see Suppl. Table 5). Other studies targeting diverse ethnic populations will have to address *LRRK2* variant dependent variance in progression.

In conclusion, we did not identify baseline predictors or longitudinal change in clinical, DAT, and biofluid biomarker characteristics in a large *LRRK2* G2019S NMC cohort. These findings are of essential importance for the research community and highlight the challenges of designing clinical trials in *LRRK2* G2019S NMCs both from the feasibility standpoint (number of participants to be screened versus the number who will qualify) and essential need for further enrichment biomarker discovery in addition to DAT deficit.

More longitudinal data and larger comprehensive biomarker panels will be necessary to identify *LRRK2* G2019S NMCs at the highest risk of progression. It also will be essential to define multimodal progression trajectories that ultimately predict the development of PD in a time period amenable to future disease prevention therapeutic interventions.

## Methods

### Study design

Data used in the preparation of this manuscript were obtained from the PPMI database (www.ppmi-info.org/data). The aims and methodology of the study have been published elsewhere^33,34^. Study protocol and manuals are available at www.ppmi-info.org/study-design. The data used for this paper were downloaded on June 30, 2020 and reflect *LRRK2* NMCs enrolled between September 2013 and May 2019 from 33 participating sites worldwide.

### Participants

The *LRRK2* NMC cohort enrolled male or female participants age 45 years or older at baseline with a *LRRK2* pathogenic variant confirmed by the Genetic Coordination Core. Participants were excluded if they had a clinical diagnosis of PD based on established diagnostic criteria^35^ or conditions that precluded safe performance of lumbar puncture. Recruitment of the NMC cohort was done via participating sites and a centralized recruitment initiative, described previously, specifically targeting first-degree relatives of PD patients of Ashkenazi Jewish descent^36^. The study was approved by the institutional review board at each site, and participants provided written informed consent. HCs were enrolled in PPMI based on previously published inclusion and exclusion criteria; notably, they were required to have a Montreal Cognitive Assessment (MoCA) score ≥27 at enrollment^34^. Due to the focus on short-term longitudinal change, we restricted analyses to NMCs and HCs who were assessed at baseline and at least one of the first two annual follow-up visits (i.e., at year 1 and/or year 2). As of the data freeze date used for this analysis, 36/176 *LRRK2* G2019S NMCs (20%) had not completed a year 2 assessment, primarily because they were not yet due for this study visit (note: only four of these participants had withdrawn).

### *LRRK2* pathogenic variants testing for the NMC cohort

Genetic analysis was performed by the PPMI genetic core. Briefly, qualified individuals underwent screening for the *LRRK2* G2019S pathogenic variants. Results were provided by phone by certified genetic counselors at Indiana University or by site-qualified personnel^36^. In addition, *LRRK2* carrier data from genetic platforms including whole genome sequencing were downloaded from the PPMI database to identify pathogenic variant carriers in the broader PPMI cohort. Our analysis focused on *LRRK2* G2019S NMCs (*N* = 176). As such, we excluded participants with other *LRRK2* pathogenic variants including R1441G due to small sample size (*N* = 16). We also excluded 19 *LRRK2* NMCs and 2 HCs who had pathogenic variants of *GBA1*. In addition, a polygenic risk score (PRS) was constructed from 89 loci associated with PD risk, which excluded the *G2019S* variant, using previously published methodology^37^.

### Study outcomes

All participants enrolled in PPMI undergo a standard test battery of assessments described in detail previously^7,8^. The clinical battery relevant to this analysis included the MDS-UPDRS, MoCA, 15-item Geriatric Depression Scale, Scale for Outcomes in PD-Autonomic (SCOPA-AUT), State and Trait Anxiety Scale (STAI), Modified Schwab and England Activities of Daily Living Scale, Questionnaire for Impulsive-Compulsive Disorders in Parkinson’s Disease (QUIP), Epworth Sleepiness Scale (ESS), RBD Screening Questionnaire (RBDSQ) and UPSIT. UPSIT was analyzed based on age/sex-specific percentiles derived from normative data. All participants are expected to undergo DATscan at baseline and then every other year to assess dopamine transporter (DAT) binding analyzed according to the PPMI imaging technical operations manual (http://ppmi-info.org/)^8^. We applied quantitative DATscan analysis using previously described methods to determine the minimum putamen specific binding ratio (SBR) and <65% age/sex-expected lowest putamen SBR was used as a cut-off for DAT deficit^34^. Of note, HCs were enrolled based on the visual assessment of DATscan (normal versus abnormal) as per regulatory approval of DATscan^38^. Visual and quantitative DAT have high but not 100% correlation^39^. Thus, some HCs had DAT deficit based on the quantitative analysis. Prodromal risk score was calculated based on previously published criteria^14^. PPMI collects an array of biofluid biomarkers (www.ppmi-info.org/access-data-specimens/specimens). Not all such measures are currently available since the analytes are measured in batches. The available biofluid biomarkers included CSF Abeta, total tau and phosphor(181)-tau; serum urate and NfL; and urine BMP. The analytical methods for monitoring these biomarkers have been published previously^7,9,18^.

### Phenoconversion

Progression to a clinically defined diagnosis of PD or other degenerative disorder (phenoconversion) is determined by the site investigator based on established diagnostic criteria. The clinical and biomarkers dataset of every phenoconverted participant is reviewed by a panel of experts (concensus committee) who adjudicate on the final diagnosis.

### Statistical methods

Statistical analyses were performed using SAS version 9.4 (SAS/STAT 15.1; SAS Institute Inc., Cary, NC) and R version 4.1.3 (R Foundation for Statistical Computing, Vienna, Austria). Baseline demographics were compared using chi-squared and *t* tests (Fisher’s exact and Wilcoxon rank-sum tests where appropriate) at α = 0.05. Cross-sectional comparisons of biofluid and imaging biomarkers, conducted using the SAS/STAT CAUSALTRT procedure, applied inverse probability weighting methods to control for age and sex. Within *LRRK2* G2019S NMCs, associations between baseline biofluids and striatal DAT SBR were assessed using Spearman partial rank-order correlations controlling for age and sex, with 95% confidence intervals (CIs) obtained using Fisher’s *z* transformation.

All models were fit using generalized estimating equations (GEE) with an autoregressive correlation structure. For most clinical and biological outcomes, group mean trajectories did not support a linear trend assumption because the outcomes in years 1 and 2 were relatively comparable. Instead, we treated year 1 and year 2 as analogous “follow-up” visits (i.e., considered them equivalent with respect to time). To compare longitudinal changes between groups, GEE with inverse probability weighting to control for age and sex, conducted using the R geepack package (because an analagous SAS procedure was unavailable), modeled the change from baseline at follow-up (continuous outcomes) or binary response at follow-up (categorical outcomes) while adjusting for the baseline value. Within *LRRK2* G2019S NMCs, the effects of baseline DAT deficit and hyposmia on longitudinal clinical changes were assessed using GEE, conducted using the SAS/STAT GENMOD procedure, modeling the change from baseline at follow-up (continuous outcomes) or binary response at follow-up (categorical outcomes) while adjusting for the baseline value. Additionally, change from baseline to follow-up in striatal DAT SBR (measured at baseline and year 2 only) and urine BMP (measured at baseline, year 1, and year 2) were assessed using one-way *t* tests and GEE, respectively.

For all applicable analyses, serum NfL and urine BMP values were log-transformed and CSF biomarkers were assigned to quintile categories or dichotomized using established cutoffs^7^. For ease of interpretation, the PRS was normalized by rescaling to mean=0 and standard deviation=1; also, analyses were adjusted for age, sex, and the first five principal components of the genetic data (representing population structure).

All statistical tests were two-sided. To account for multiple comparisons, we applied a family-wise error rate to each set of analyses. Specifically, a Bonferroni correction, computed as 0.05/number of family-wise hypotheses tested per table, was applied to Tables 2–5 and applicable supplementary tables.

## Supplementary information

Supplementary Tables

## Data Availability

PPMI is an open access dataset. Data used in the preparation of this manuscript were obtained from the PPMI database (www.ppmi-info.org/data). Study protocol and manuals are available at www.ppmi-info.org/study-design. The data used for this paper were downloaded on June 30, 2020.
