# Supplementary material for: Longitudinal clinical and biomarker characteristics of non-manifesting LRRK2 G2019S carriers in the PPMI cohort
Source: NPJ Parkinsons Dis. 2022 Oct 22;8:140. doi: 10.1038/s41531-022-00404-w (PMC9588016; doi:10.1038/s41531-022-00404-w)
Supplement: Supplementary file 1 — Supplementary Tables [file 41531_2022_404_MOESM1_ESM.pdf]

Supplementary Table 1. Change in clinical characteristics among *LRRK2* G2019S carriers with *vs* without baseline hyposmia

	***LRRK2* G2019S carriers: baseline hyposmia**			***LRRK2* G2019S carriers: no baseline hyposmia**			**p value**
	Baseline (N = 20)	Year 1 (N = 20)	Year 2 (N = 13)	Baseline (N = 156)	Year 1 (N = 152)	Year 2 (N = 127)	Group × time effect
**MDS-UPDRS total score**	11.2 (11.3)	11.4 (11.7)	12.1 (14.7)	8.6 (6.9)	9.4 (7.9)	9.1 (8.5)	0.6752
**MDS-UPDRS Part I**	5.0 (5.0)	5.5 (5.8)	5.0 (5.6)	4.8 (3.7)	5.0 (3.9)	4.7 (3.9)	0.8862
**MDS-UPDRS Part II**	2.3 (3.9)	2.9 (5.4)	2.7 (4.1)	1.0 (1.9)	1.2 (2.2)	1.3 (2.4)	0.3978
**MDS-UPDRS Part III**	4.7 (5.9)	4.2 (3.9)	3.8 (5.3)	2.9 (3.8)	3.1 (4.1)	3.2 (4.9)	0.6363
**MOCA total score**, mean (SD; range)	26.9 (2.3; 22-30)	26.8 (2.6; 22-30)	27.2 (2.3; 23-30)	26.8 (2.5; 18-30)	27.1 (2.5; 16-30)	26.9 (2.5; 20-30)	0.9816
**GDS-15 total score**	2.2 (3.9)	1.9 (3.7)	2.5 (4.6)	1.7 (2.2)	1.5 (1.8)	1.6 (2.0)	0.9198
**SCOPA-AUT total score**	10.8 (6.2)	11.2 (8.5)	11.3 (7.0)	8.2 (6.0)	8.5 (6.3)	8.3 (6.1)	0.4918
**Orthostatic systolic blood pressure drop**	-3.1 (12.8)	-0.2 (11.2)	-1.3 (7.8)	0.5 (10.7)	-1.0 (11.2)	-0.4 (10.9)	0.7726
**Orthostatic diastolic blood pressure drop**	-5.2 (6.4)	-3.9 (7.8)	-2.3 (7.0)	-3.5 (7.8)	-4.7 (7.6)	-3.3 (7.6)	0.4713
**State trait anxiety score**	64.9 (18.6)	63.0 (22.6)	70.9 (24.2)	60.9 (16.5)	59.8 (15.8)	59.5 (15.8)	0.5323
**QUIP (≥ 1 disorder)**	5 (28%)	5 (29%)	3 (27%)	41 (26%)	40 (27%)	41 (33%)	0.8616
**Epworth sleepiness scale (≥ 10)**	2 (11%)	4 (22%)	3 (27%)	16 (10%)	13 (9%)	11 (9%)	0.0686
**RBDSQ (≥ 5)**	2 (12%)	4 (25%)	1 (9%)	36 (23%)	31 (21%)	26 (21%)	0.6986

Data are mean (SD) or n (%) unless otherwise stated. Hyposmia defined by UPSIT ≤ 15th percentile for age and sex. Five or fewer participants per group missed any one assessment except for the non-hyposmia group at year 2 (seven missed the SCOPA-AUT). p values were found using generalized estimating equations modeling the change from baseline at follow-up (continuous outcomes) or the binary response at follow-up (categorical outcomes) while adjusting for the baseline value. Significance level for comparisons is $p < 0.0038$ (after Bonferroni correction). UPSIT=University of Pennsylvania Smell Identification Test. MDS-UPDRS=Movement Disorder Society Unified Parkinson's Disease Rating Scale. MOCA=Montreal Cognitive Assessment. GDS-15=Geriatric Depression Scale (15-item). SCOPA-AUT=Scales for Outcomes in Parkinson's Disease-Autonomic. QUIP=Questionnaire for Impulsive-Compulsive Disorders in Parkinson's Disease. RBDSQ=REM Sleep Behavior Disorder Screening Questionnaire.

Supplementary Table 2. Change in DAT specific binding ratios among *LRRK2* G2019S carriers and comparison to healthy controls at baseline

	***LRRK2* G2019S carriers**		**Healthy controls**	**p values**	
	Baseline (N = 176)	Year 2 (N = 140)	Baseline (N = 185)	Time effect	Group effect
**Caudate SBR**	2.97 (0.57; 1.38-4.30)	2.89 (0.61; 1.27-4.59)	2.99 (0.61; 1.56-5.20)	0.1929	0.0520
**Putamen SBR**	2.09 (0.51; 0.67-3.70)	2.03 (0.54; 0.89-3.23)	2.15 (0.54; 0.88-3.89)	0.0865	0.0237
**Striatum SBR**	2.53 (0.52; 1.17-4.00)	2.46 (0.56; 1.12-3.85)	2.57 (0.55; 1.22-4.24)	0.0910	0.0292

Data are mean (SD; range). DAT imaging results were missing for 8 *LRRK2* G2019S carriers at baseline, 18 *LRRK2* G2019S carriers at year 2, and 1 healthy control at baseline. p values were found using one-sample t-tests assessing the percent change from baseline to year 2 among *LRRK2* G2019S carriers (time effect) and inverse probability weighting methods comparing *LRRK2* G2019S at year 2 vs healthy controls at baseline while adjusting for age and sex (group effect). Significance level for comparisons is $p < 0.0167$ (after Bonferroni correction). DAT=dopamine transporter. SBR=specific binding ratio.

Supplementary Table 3. Change in urine bis(monoacylglycerol)phosphate among *LRRK2* G2019S carriers

	***LRRK2* G2019S carriers**			***p* value**
	Baseline (N = 176)	Year 1 (N = 172)	Year 2 (N = 140)	Time effect
**Total di-18:1-BMP**	17.5 (13.4)	18.6 (17.0)	20.3 (17.7)	0.1523
Median (min, max)	14.2 (0.9, 72.0)	13.0 (2.3, 99.0)	14.8 (1.7, 76.5)	
**Total di-22:6-BMP**	77.3 (46.5)	81.0 (52.8)	85.5 (55.2)	0.3304
Median (min, max)	63.8 (10.6, 245.6)	67.2 (7.5, 289.6)	72.6 (15.8, 320.8)	
**2,2'-di-22:6 BMP**	61.3 (40.3)	61.9 (42.5)	65.9 (42.9)	0.8168
Median (min, max)	49.6 (8.1, 248.2)	47.0 (2.2, 244.2)	57.1 (12.8, 252.8)	
Missing BMP	33	32	54	

Data are mean (SD). p values were found using generalized estimating equations modeling the change from baseline at follow-up. Significance levels for comparisons are $p < 0.0167$ (after Bonferroni correction).
BMP= bis(monoacylglycerol)phosphate.

Supplementary Table 4. Change in clinical characteristics among *LRRK2* G2019S carriers who phenoconverted *vs* those who did not

	***LRRK2* G2019S carriers: phenoconverters**			***LRRK2* G2019S carriers: non-converters**		
	Baseline (N = 5)	Year 1 (N = 5)	Year 2 (N = 3)	Baseline (N = 171)	Year 1 (N = 167)	Year 2 (N = 137)
**MDS-UPDRS total score**	22.0 (12.7)	24.2 (11.7)	35.0 (19.3)	8.5 (6.9)	9.1 (7.9)	8.8 (8.1)
**MDS-UPDRS Part I**	6.4 (5.5)	6.0 (2.9)	7.7 (2.5)	4.7 (3.8)	5.0 (4.2)	4.7 (4.1)
**MDS-UPDRS Part II**	5.4 (3.4)	5.2 (3.2)	6.0 (2.6)	1.0 (2.1)	1.3 (2.6)	1.3 (2.5)
**MDS-UPDRS Part III**	10.2 (6.3)	13.0 (7.2)	21.3 (15.8)	2.9 (3.8)	2.9 (3.6)	2.8 (3.7)
**MOCA total score**, mean (SD; range)	25.5 (2.6; 23-29)	25.5 (1.0; 25-27)	25.7 (0.6; 25-26)	26.8 (2.4; 18-30)	27.1 (2.5; 16-30)	26.9 (2.4; 20-30)
**GDS-15 total score**	1.4 (1.7)	1.2 (1.3)	0.7 (1.2)	1.7 (2.4)	1.5 (2.1)	1.7 (2.3)
**SCOPA-AUT total score**	10.6 (7.7)	10.4 (4.7)	12.3 (2.5)	8.4 (6.0)	8.7 (6.6)	8.5 (6.3)
**Orthostatic systolic blood pressure drop**	-2.0 (9.9)	1.0 (12.7)	-2.0 (7.8)	0.2 (11.0)	-0.9 (11.1)	-0.4 (10.7)
**Orthostatic diastolic blood pressure drop**	-0.6 (5.0)	-5.2 (4.3)	-4.7 (5.5)	-3.8 (7.7)	-4.6 (7.7)	-3.2 (7.6)
**State trait anxiety score**	64.6 (21.0)	58.8 (12.8)	58.7 (9.0)	61.3 (16.6)	60.2 (16.7)	60.5 (16.9)
**QUIP (≥ 1 disorder)**	0 (0%)	0 (0%)	1 (33%)	46 (27%)	45 (28%)	43 (32%)
**Epworth sleepiness scale (≥ 10)**	0 (0%)	0 (0%)	0 (0%)	18 (11%)	17 (10%)	14 (11%)
**RBDSQ (≥ 5)**	0 (0%)	1 (20%)	0 (0%)	38 (23%)	34 (21%)	27 (20%)

Data are mean (SD) or n (%) unless otherwise stated. Among phenoconverters, there were no missing values except for the MOCA (one missing value at baseline and year 1); among non-converters, five or fewer participants missed any one assessment except for at year 1 (six missed the MDS-UPDRS total score and RBDSQ) and year 2 (10 missed the SCOPA-AUT; six missed the STAI). MDS-UPDRS=Movement Disorder Society Unified Parkinson's Disease Rating Scale. MOCA=Montreal Cognitive Assessment. GDS-15=Geriatric Depression Scale (15-item). SCOPA-AUT=Scales for Outcomes in Parkinson's Disease-Autonomic. QUIP=Questionnaire for Impulsive-Compulsive Disorders in Parkinson's Disease. RBDSQ=REM Sleep Behavior Disorder Screening Questionnaire.

Supplementary Table 5. Demographics and baseline characteristics of *LRRK2* G2019S *vs* *LRRK2* R1441G carriers

	Group		p values
	***LRRK2* G2019S carriers** (N = 176)	***LRRK2* R1441G carriers** (N = 16)	***LRRK2* G2019S *vs LRRK2* R1441G**
**Sex (female)**	99 (56%)	10 (63%)	0.7935
**Age**, mean (SD; range)	62.0 (7.7; 45-81)	56.4 (5.2; 49-63)	0.0035
**Education (≤ 12 years)**	26 (15%)	6 (38%)	0.0312
**Ethnicity (Hispanic/Latino)**	15 (9%)	14 (88%)	<.0001
**Race (White)**	171 (98%)	5 (31%)	<.0001
**Family history of PD (first-degree)**	152 (86%)	16 (100%)	0.2286
**UPSIT raw score**, mean (SD; range)	33.1 (4.3; 14-40)	29.9 (2.6; 24-34)	<.0001
**UPSIT percentile**			<.0001
≤ 15th	20 (11%)	8 (50%)	
16th-50th	73 (41%)	8 (50%)	
> 50th	83 (47%)	0 (0%)	
**DAT deficit**	21 (13%)	3 (19%)	0.4437
Missing DAT results	8	0	
**Caudate SBR**	2.97 (0.57)	3.10 (0.73)	0.7824
**Putamen SBR**	2.09 (0.51)	2.15 (0.64)	0.2469
**Striatum SBR**	2.53 (0.52)	2.63 (0.66)	0.5036
**MDS prodromal criteria (> 80%)**	12 (7%)	0 (0%)	0.6036

Data are mean (SD) or n (%) unless otherwise stated. DAT deficit defined as < 65% age/sex-expected lowest putamen SBR. Ethnicity and race were missing for one *LRRK2* G2019S subject. p values for SBR measures were adjusted for age and sex using inverse probability weighting; all other p values were found using Fisher's exact and Wilcoxon rank-sum tests. UPSIT=University of Pennsylvania Smell Identification Test. DAT=dopamine transporter. SBR=specific binding ratio. MDS= International Parkinson and Movement Disorder Society.
